# Managing Dialysis-Associated Haemodynamic Instability in Critical Calcific Left Main Stem Disease With Tachycardia-Bradycardia Syndrome Using Intravascular Lithotripsy-Assisted Percutaneous Coronary Intervention and Permanent Pacemaker Implantation

**DOI:** 10.7759/cureus.96545

**Published:** 2025-11-11

**Authors:** Claire Ng, Adrian Ng Ping Vey, Keng Tian Ng, Kin Sing Fan

**Affiliations:** 1 Hospital Medicine, Aberdeen Royal Infirmary, Aberdeen, GBR; 2 School of Medicine, University of Manchester, Manchester, GBR; 3 Cardiology, Gleneagles Hospital Kuala Lumpur, Kuala Lumpur, MYS; 4 Nephrology, Gleneagles Hospital Kuala Lumpur, Kuala Lumpur, MYS

**Keywords:** bradycardia-tachycardia syndrome, complex percutaneous coronary intervention, end stage renal disease (esrd), esrd with extensive vascular calcification, haemodialysis (hd), intravascular lithotripsy, left main stem (lms), oct angiography, permanent pacemaker implantation (ppm), sinoatrial node dysfunction

## Abstract

Patients with end-stage renal disease experience an accelerated form of systemic vascular calcification involving both intimal atherosclerosis and medial calcific sclerosis (Mönckeberg's sclerosis). This dual pathology creates unique challenges for coronary revascularisation.

We present a dialysis-dependent woman with severe calcific critical left main stem bifurcation disease who underwent successful intravascular lithotripsy-assisted, optical coherence tomography-guided percutaneous coronary angioplasty. She had concomitant severe bradycardia from sinoatrial node dysfunction with paroxysmal atrial fibrillation, which resolved with implantation of a pacemaker. This case highlights the pathobiology underpinning dysmorphic vascular calcification in end-stage renal disease, its impact upon the complex cardiac presentation, and the need for a specialised device-based strategy to achieve a favourable outcome.

## Introduction

Patients with end-stage renal disease (ESRD) on haemodialysis have a 10- to 20-fold higher cardiovascular mortality compared to the general population of similar age and sex [1]. Based on the United States Renal Data System (USRDS) database, cardiovascular disease accounts for nearly 43% of all deaths in this group, of which 27% are due to sudden cardiac death (SCD) [2]. Atrial fibrillation occurs in up to 20-25% of dialysis patients compared with 2-3% in the general population, while bradyarrhythmias requiring pacemaker implantation are several-fold more prevalent [3,4].

A major driver of this epidemic of cardiovascular disease is calcification, i.e., atherosclerotic intimal calcification, arterial medial calcification, and calcific aortic stenosis [5]. Budoff et al. demonstrated that CAC (coronary artery calcium) scoring by electron-beam CT predicted mortality and cardiovascular events more accurately than any serum biomarker [6].

We present an emergent case of severe calcific critical left main stem (LMS) bifurcation disease involving the proximal left anterior descending (LAD) and circumflex arteries (Cx), with coexisting paroxysmal atrial fibrillation (PAF) and sinus bradycardia (tachycardia-bradycardia syndrome) in a dialysis-dependent patient, and discuss how the underlying pathophysiology impacted the clinical presentation as well as the eventual management.

## Case presentation

A 78-year-old housewife with a 28-year history of diabetes mellitus and hypertension presented in March 2023 with palpitations and rest angina, accompanied by severe dyspnoea and dizziness midway through her thrice-weekly haemodialysis session at the local hospital. Eight years previously, she developed ESRD and has since been on calcium carbonate, simvastatin, felodipine, trimetazidine, folic acid, vitamin B complex, bisoprolol, and Mixtard insulin.

**Figure 1:**
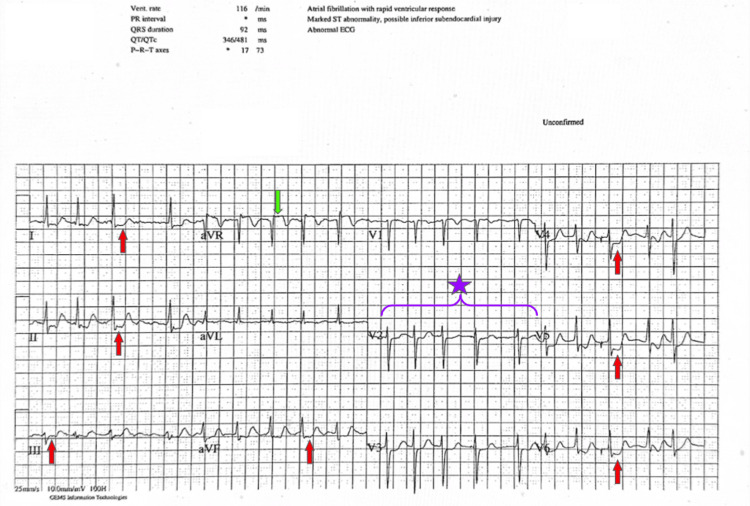
Intradialysis ECG showing PAF (region marked by a purple curly brace with a purple star), ST depression in leads I–III, aVF, and V4–V6 (red arrows), and ST elevation in lead aVR (green arrow), indicating global subendocardial ischaemia. PAF: paroxysmal atrial fibrillation.

Following admission to a tertiary hospital, echocardiography demonstrated concentric left ventricular hypertrophy, diastolic dysfunction, an enlarged left atrium, and a preserved ejection fraction of 55%, with no obvious regional wall motion abnormality. During dialysis, the patient had a recurrence of her symptoms. ECG confirmed intradialytic paroxysmal atrial fibrillation (PAF) with transient widespread ST depression, indicating diffuse subendocardial ischaemia (Figure 1).

**Figure 2:**
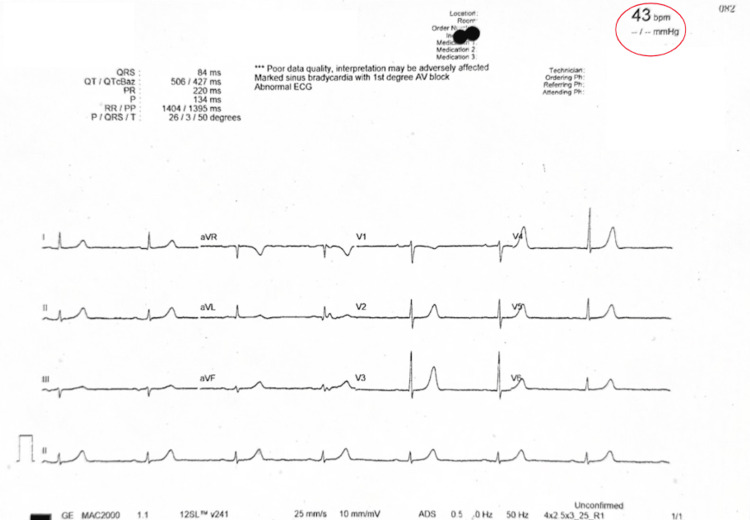
Intra-dialysis ECG showing severe sinus bradycardia at 43 bpm (encircled in red).

**Figure 3:**
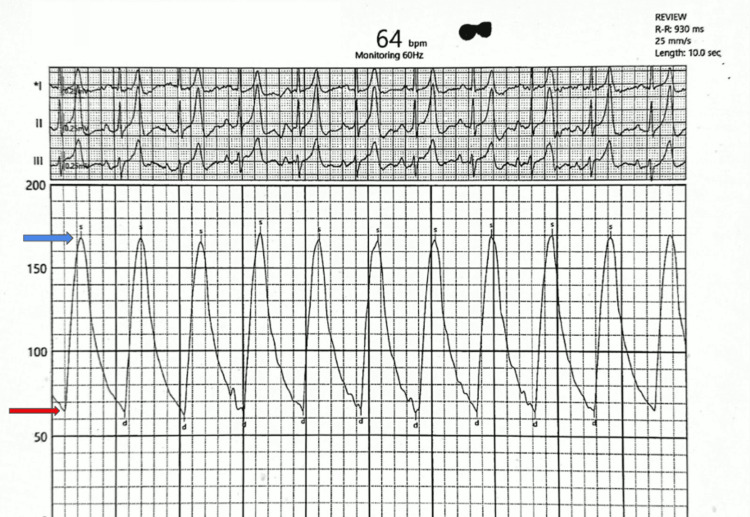
Intra-aortic pressure recording during angiography. Pulse pressure of 104 mmHg with elevated systolic pressure of 168 mmHg (blue arrow) and diastolic pressure of 64 mmHg (red arrow). Pulse pressure = systolic pressure - diastolic pressure.

Upon spontaneous cessation of the PAF, severe sinus bradycardia (Figure 2) followed. This was consistent with a diagnosis of tachycardia-bradycardia syndrome. Manual blood pressure was elevated at 170/65 mmHg, with a marked elevation in pulse pressure confirmed later during coronary angiography (Figure 3).

Coronary angiography demonstrated severe calcific stenosis of the LMS bifurcation involving both the LAD and Cx vessels, with a "tram-track" appearance of both vessels indicating extensive calcification of the LAD and Cx arteries. She was then referred for bypass surgery. Efforts to schedule her with several different cardiac surgical units were unsuccessful.

After careful deliberation of the pros and cons of both rotational atherectomy (RA) and intravascular lithotripsy (IVL), a decision was made to proceed with IVL-assisted percutaneous transluminal coronary angioplasty (PTCA), considering the high-risk coronary anatomy with ongoing ischaemia. Both LAD and Cx arteries were wired, and optical coherence tomography (OCT) was performed using normal saline instead of contrast injection. A 3 mm IVL balloon was then deployed at 4 atmospheres at the LAD stenosis. Unfortunately, it was punctured by a spicule of calcific plaque after an initial 10 pulses. This was overcome with a 3.5 mm IVL balloon, which allowed dilation of the LAD and distal LMS after delivery of 40 pulses. The same balloon was then deployed at the proximal Cx artery to deliver the remaining 40 pulses.

**Figure 4:**
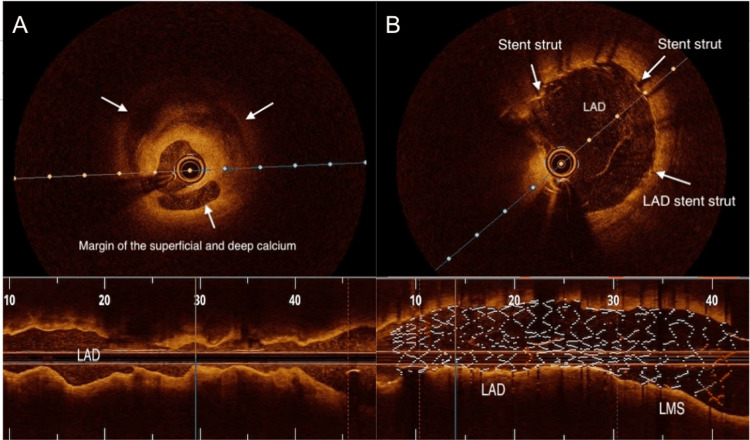
OCT images of the LAD Minimal lumen diameter of approximately 1.5 mm before IVL-assisted stenting (A) and post-stenting minimum stent diameter of 3.5-3.75 mm after IVL-assisted stenting (B). In each pane, white text annotations correspond to the regions highlighted by white arrows. OCT: optical coherence tomography; LAD: left anterior descending artery; IVL: intravascular lithotripsy.

**Figure 5:**
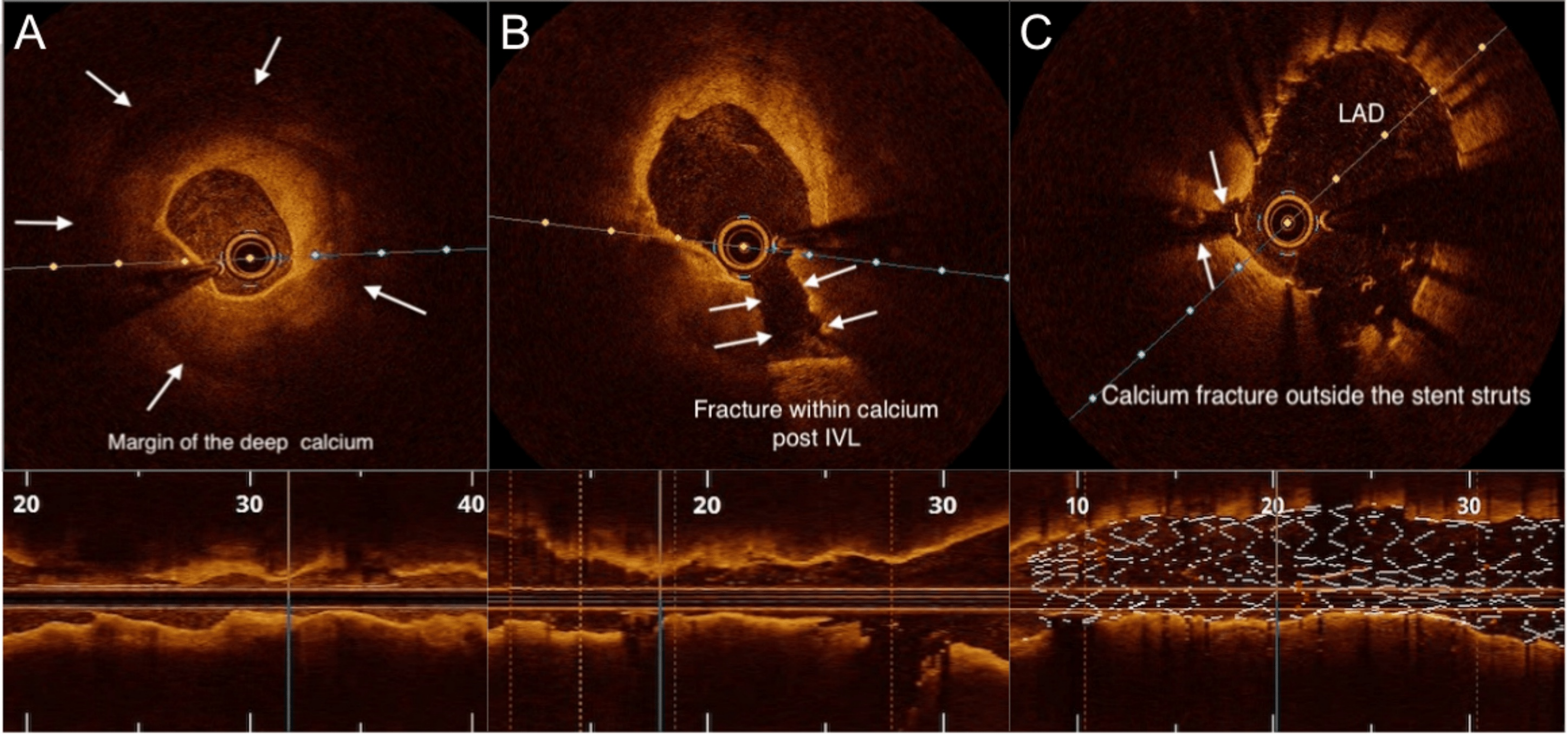
OCT images of the LAD Minimum lumen diameter of approximately 1.5mm before IVL (A) and fracture of the calcium plate after IVL (B).  Post-stenting minimum stent diameter of approximately 3.75 mm after final stent implantation (C). In each pane, white text annotations correspond to the regions highlighted by white arrows. OCT: optical coherence tomography; LAD: left anterior descending artery; IVL: intravascular lithotripsy.

**Figure 6:**
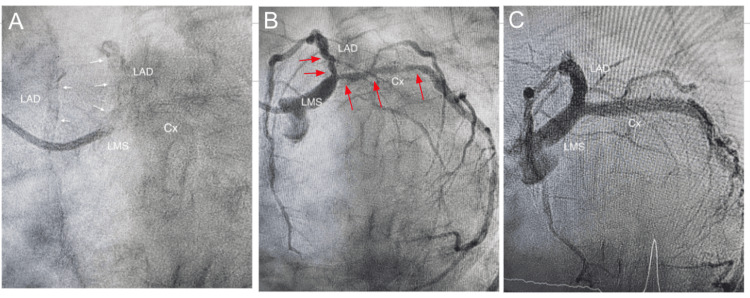
Angiographic LAO-caudal views Dry cine run of the LMS, LAD, and Cx arteries, which are heavily lined with parallel calcium deposits on both sides, i.e, tramtrack calcification (white arrows) (A). Severe stenosis of distal LMS, LAD, and Cx arteries (red arrows) (B). Post-PCI results with minimal residual stenosis of the ostial Cx (C). LAO: left anterior oblique; LMS: left main stem; LAD: left anterior descending artery; Cx: circumflex artery; PCI: percutaneous coronary Iintervention.

**Figure 7:**
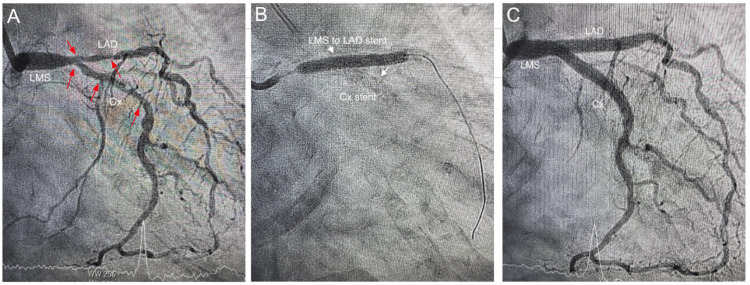
Angiographic RAO-caudal views Severe stenosis of distal LMS, LAD, and Cx arteries marked by red arrows (A). Deployment of LMS to LAD stent after successfully deploying the Cx stent, both stents marked by white arrows (B). Post-PCI results (C). RAO: right anterior oblique; LMS: left main stem; LAD: left anterior descending artery; Cx: circumflex artery; PCI: percutaneous coronary intervention.

A 3.5 mm × 32 mm drug-eluting stent (DES) was then deployed at the Cx artery, followed by a 4 mm × 32 mm DES to cover the LMS and proximal LAD. A kissing balloon dilation restored the carina and the polygon of confluence of the distal LMS. The procedure was completed after final dilation of the LMS with a 5 mm balloon. Post-procedural OCT runs and angiography confirmed adequate stent expansion and strut apposition (Figures 4A-7C). 

The immediate post-procedure recovery period was punctuated by an episode of hypotension to 70 mmHg, associated with a seizure. The neurologist who reviewed her attributed this event to cerebral hypoxia exacerbated by hypotension resulting from intra-procedural blood loss and an evolving large subcutaneous haematoma of the anterior abdominal wall due to repeated insulin injections. Post-procedural haemoglobin levels dropped to 6 g/dL and improved to 9.5 g/dL with a saline infusion followed by judicious transfusion of packed cells during her subsequent two dialysis sessions.

**Figure 8:**
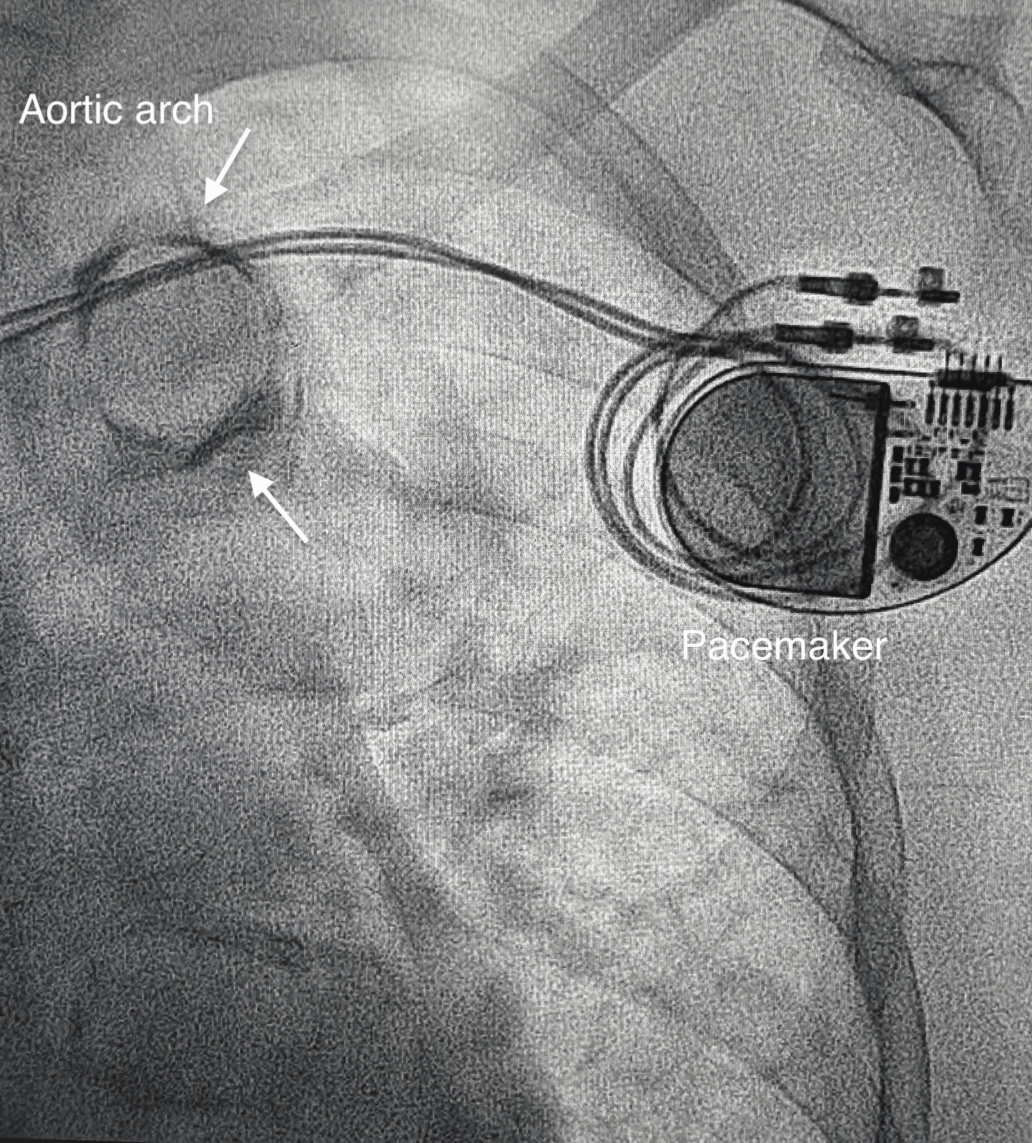
DDDR pacemaker in-situ Notable heavy calcification of the aortic arch "Mönckeberg's sclerosis" (white arrow) was observed.

Despite this, the patient continued to experience palpitations from PAF and bradycardia. This was resolved with the implantation of a DDDR pacemaker a week later (Figure 8).

Her recovery was uneventful, and she was discharged to continue her haemodialysis sessions at the district hospital. At 30 months’ follow-up, she remained fully mobile and in sinus rhythm, with no recurrence of palpitations or angina.

## Discussion

Pathobiology of arterial calcification

Atherosclerosis starts early in life and remains asymptomatic through the initial stages of pathological intimal hyperplasia and fibroatheroma [7,8]. Later, haemorrhage from the vasa vasorum leads to the formation of a necrotic core and eventual plaque rupture [9]. This manifests as angina, myocardial infarction, or SCD. SCD is secondary to an occlusive thrombus from a plaque rupture in 60-65% of cases, plaque erosion in 20-30%, and an eruptive calcium nodule in 2% [10]. Remarkably, in this patient, the disease manifested clinically in later life.

In medial calcification, the contractile vascular smooth muscle cells (VSMCs) undergo osteogenic differentiation under the influence of hyperphosphataemia, secondary hyperparathyroidism, and advanced glycation end products, which activate the transcription factor for osteoblastic differentiation, RunX2. Transdifferentiated VSMCs express bone proteins such as alkaline phosphatase and bone Gla-protein (osteocalcin) while losing calcification inhibitors such as matrix Gla-protein, pyrophosphate, and fetuin-A. This results in hydroxyapatite deposition, eventually leading to true osseous metaplasia [11,12]. Normally, the proximal aorta dampens pressure oscillations from ventricular systole (Windkessel effect) and releases stored energy during diastole to transform the pulsatile flow of arteries into a steady peripheral flow. Elastolysis, collagen deposition, and medial calcification increase aortic wall stiffness. The resultant elevated pulse wave velocity leads to a reflected wave during systole rather than diastole, raising systolic and lowering diastolic pressure, which then widens the pulse pressure, as exemplified in this patient.

With ventricular hypertrophy from systolic hypertension and critical LMS bifurcation disease, this patient developed angina with widespread ST depression when stressed by PAF. Loss of atrial systole, which contributes up to 30-40% of left ventricular filling, led to an increase in left atrial pressure and pulmonary congestion, hence her marked dyspnoea.

Arrhythmias in ESRD

In our patient, symptomatic tachycardia-bradycardia syndrome necessitated pacemaker implantation to allow the safe continuation of dialysis. Tachycardia-bradycardia syndrome (also known as sick sinus syndrome with alternating bradyarrhythmia and tachyarrhythmia) is a manifestation of sinoatrial node dysfunction characterised by periods of inappropriate sinus bradycardia, sinus arrest, or sinoatrial block alternating with episodes of supraventricular tachyarrhythmias, most commonly PAF or atrial flutter. Clinically, patients may present with palpitations, dizziness, syncope, or heart failure symptoms due to fluctuating cardiac output. This coexistence reflects shared mechanisms: chronic atrial stretch from volume overload, atrial fibrosis, autonomic instability from fluid shifts, and electrolyte fluctuations (especially potassium and calcium) [2,5]. Sinoatrial node dysfunction is potentiated by fibrosis, vascular calcification, microvascular ischaemia, and autonomic imbalance [12].

PAF terminates spontaneously, allowing the sinoatrial node dysfunction to manifest as a prolonged pause, often with concomitant dizziness or syncope before resumption of sinus rhythm. In our case, the use of beta-blockade to control the ventricular rate of PAF worsened the sinus node dysfunction, resulting in bradycardia of up to 43 bpm, as seen in Figure 2. Ultimately, this was resolved with the implantation of a DDDR pacemaker [13].

PTCA of heavily calcified coronaries

PTCA of calcified lesions has a poorer outcome compared with non-calcified lesions [14]. Imaging with OCT or intravascular ultrasound (IVUS) has been shown to reduce adverse events [15,16].

OCT is an intravascular imaging modality that utilises near-infrared light to obtain high-resolution cross-sectional images of the coronary vessel wall, enabling accurate assessment of calcium length, thickness, and arc, as well as lumen dimensions, stent apposition, and vessel healing [17,18]. Owing to the complexity of the case, OCT was chosen for its superior axial resolution (10-20 µm compared with approximately 60 µm for IVUS) [19], facilitating comprehensive lesion evaluation and procedural optimisation throughout the PTCA.

IVL-assisted PTCA is an advanced technique used to facilitate stent deployment in heavily calcified coronary lesions. IVL employs acoustic pressure waves, delivered via a specialised balloon catheter, to fracture both superficial and deep calcium deposits within the vascular wall, thereby improving vessel compliance and enabling optimal lesion expansion and stent apposition [20,21]. The IVL generator delivers 3 kV of energy to emitters within the balloon once per second, vaporising the surrounding fluid and creating a rapidly expanding bubble that produces a sonic pressure wave lasting only a few microseconds [22]. When these waves impact calcified plaque, at pressures reaching up to 50 atmospheres, they generate a series of microfractures within both superficial and deep calcium layers, rendering the vessel more compliant and allowing safe stent delivery [20,22].

Unlike traditional rotational atherectomy, IVL acts circumferentially with minimal vessel trauma, significantly reducing the risk of dissection, distal embolisation, or the "no-reflow" phenomenon, which can result from microvascular spasm or debris embolisation during plaque ablation [21,23]. The procedure is typically performed after lesion crossing with a guidewire and accurate balloon positioning, followed by activation of lithotripsy pulses under low-pressure balloon inflation [20].

Clinical alternatives 

Coronary artery bypass graft (CABG) surgery carries a peri-operative mortality risk of 13-15%. Among those successfully operated upon, the 5-year survival rate is 41% [24,25]. Operating on heavily calcified coronaries is challenging, and manipulation of a heavily calcified aorta increases the risk of systemic atheroembolism. The likelihood of peri-operative bleeding and infection is elevated, while the management of fluids, electrolytes, and haemodynamics is complicated.

Of note, statins have failed to reduce vascular calcification [26]. The 4D, AURORA, and SHARP trials showed no mortality benefit of statins in dialysis or CKD patients [27-29]. Statins, by inhibiting the synthesis of isoprenoids via the mevalonate pathway, disrupt macrophage Rac1 regulation, thereby paradoxically accelerating calcification [30]. Similarly, vitamin K antagonists such as warfarin are associated with increased vascular calcification, osteoporosis, and the uncommon but devastating calcific uraemic arteriolopathy (calciphylaxis).

Arrhythmia burden and SCD in ESRD

Alpert, in his comprehensive review [31], highlighted the prognostic impact of arrhythmias, with SCD accounting for nearly one-third of deaths in haemodialysis patients. Lethal arrhythmias, rather than ischaemia, were the predominant mechanism. Nevertheless, a major randomised controlled trial of prophylactic implantable cardioverter-defibrillator (ICD) implantation in patients with a left ventricular ejection fraction of at least 35% was discontinued for lack of efficacy [32].

This category of death deserves special attention, as most victims were still leading productive lives. It may be prudent to avoid calcium-containing phosphate binders [33] and low-potassium dialysate, while maintaining an optimal pre-dialysis serum potassium level [34]. Beta-blockers and ACE inhibitors should be preferred for hypertension. Finally, dialysis units should be equipped with a defibrillator, and staff trained in advanced cardiac life support to manage cardiac arrests.

## Conclusions

This case typifies the convergence of decades of exposure to diabetes, hypertension, ageing, sedentary lifestyle, and chronic kidney disease that characterise our present populace, leading to widespread dystrophic vascular calcification.

Population-wide primary prevention measures addressing these modifiable risk factors have proven highly effective. However, more research into effective measures of secondary prevention to improve long-term survival is needed. A coordinated effort between the nephrologist, dialysis unit staff, and the timely involvement of the cardiologist, electrophysiologist, and endocrinologist to manage emerging problems may help prolong the longevity of this unique population, as exemplified by this patient.
